# Comparison of IL-17 and FOXP3+ Levels in Maternal and Children Leprosy Patients in Endemic and Nonendemic Areas

**DOI:** 10.1155/2021/8879809

**Published:** 2021-02-24

**Authors:** Flora Ramona Sigit Prakoeswa, Faradiba Maharani, Munawaroh Fitriah, Jusak Nugraha, Hardyanto Soebono, Budi Prasetyo, Santi Martini, Dominicus Husada, Hari Basuki Notobroto, Muhammad Yulianto Listiawan, Anang Endaryanto, Cita Rosita Sigit Prakoeswa

**Affiliations:** ^1^Doctoral Program, Faculty of Medicine, Airlangga University, Surabaya, Indonesia; ^2^Dermatology and Venereology Department, Faculty of Medicine, Universitas Muhammadiyah Surakarta, Surakarta, Indonesia; ^3^Faculty of Medicine, Sebelas Maret University, Surakarta, Indonesia; ^4^Department of Clinical Pathology, Faculty of Medicine, Airlangga University, Dr. Soetomo General Academic Hospital, Surabaya, Indonesia; ^5^Department of Dermatology and Venereology, Faculty of Medicine, Public Health and Nursing, Gadjah Mada University, Yogyakarta, Indonesia; ^6^Department of Obstetrics and Gynecology, Faculty of Medicine, Airlangga University, Dr. Soetomo General Academic Hospital, Surabaya, Indonesia; ^7^Faculty of Public Health, Airlangga University, Surabaya, Indonesia; ^8^Department of Pediatric, Faculty of Medicine, Airlangga University, Dr. Soetomo General Academic Hospital, Surabaya, Indonesia; ^9^Department of Dermatology and Venereology, Faculty of Medicine, Airlangga University, Dr. Soetomo General Academic Hospital, Surabaya, Indonesia

## Abstract

Leprosy, a chronic infection caused by *M. leprae*, has a complex transmission problem that makes eradication programs difficult. New cases and ongoing transmission of leprosy in endemic areas make individuals living in endemic environments vulnerable to leprosy. This can be caused by the dysregulation of immune system in individuals living in leprosy-endemic areas. Although the number of male leprosy patients is higher, female leprosy patients have more impact on the family health status due to close contact with family members, roles in the household, and parenting. This could cause the increased number of children leprosy patients. We investigated the dysregulation of immune system by comparing IL-17 and FOXP3+ levels occurring in maternal and child leprosy patients in endemic and nonendemic areas. The results of the study found a statistically significant difference in IL-17 levels between the MB leprosy patient group and the control group (*p*=0.048), where higher levels of IL-17 are observed in the control group. A significant difference also was found in FOXP3+ levels between the group of healthy children living in endemic and those living in nonendemic areas (*p*=0.047), where higher FOXP3+ is observed in the healthy children living in endemic areas group.

## 1. Introduction

Leprosy is a chronic infection caused by acid-resistant bacteria, *Mycobacterium leprae* [1]. Leprosy complex problems in transmission contributed to the unfinished eradication program and left leprosy-endemic areas existed. According to the latest WHO data in 2020, there were 177,175 registered cases of leprosy with a prevalence rate 22.7 per million population [2]. The highest new leprosy cases were found in India with 114,451 cases, followed by Brazil with 27,863 cases and Indonesia with 17,439 cases, where 1,121 among the new cases are with grade 2 disability (G2D) [2].

Almost all eastern part provinces of Indonesia are areas with high leprosy burden. According to the newest health profile of Indonesia in 2019, East Java Province holds the highest number of leprosy cases in the western part of Indonesia (Sumatera and Java Island) with 2,940 cases and the G2D rate 8.06 per million population. Moreover, the total number of female leprosy new cases is 1,150 (39.12%) and pediatric leprosy cases are 202 (6.87%) [3].

The relatively stable number of new cases found in endemic areas possibly caused by the failure to break the transmission chain [4]. The termination of leprosy transmission chain depends on several aspects such as the characteristics of bacteria (microbiological aspects), host (immunological aspects), and environmental factors [4, 5].

Individuals lived in endemic environment become more susceptible to leprosy due the dysregulation of immune system which caused easier transmission of *M. leprae*. Although sex distribution of leprosy patients shows males are more affected than females, a previous study in Indonesia showed that female patients have been more affected due to stigma and discrimination towards leprosy disease [6]. Women health, especially those in childbearing age, could affect the family health conditions. The dominant role of women on taking care of their family increases the chance of females with leprosy to transmit the disease to other house members, especially their children. During pregnancy, women who experienced infection, malnutrition, obesity, and exposure to cigarette smoke could induce the immune dysregulation of her children [7]. Moreover, females in developing countries tend to get late health treatment in healthcare for any health-related problems [8]. Failure to resolve the causes of dysregulation immune system in communities that live in leprosy endemic environments makes transmission of *M. leprae* bacteria easier since the host becomes more susceptible to leprosy [9]. In contrast to nonendemic areas, new cases of leprosy were not found and associated with a good immune system condition [10]. The enhancement of the immune system is carried out gradually since the beginning of life, one of which is through perinatal health status which is influenced by the environment.

There are four cells that play roles in the body's immune response to leprosy, namely, through Th1, Th2, Treg, and Th17 cells [9]. Th1 and Th2 had been widely studied to be associated with immune system against leprosy. Defect in Th1 and Th2 response was believed to play roles in the difference of leprosy clinical manifestation. However, recent studies have explained the possibility of other subsets of T cell, like Treg and Th17 [11–13].

Previously, Treg cells role had known to keep the balance of Th1 and Th2 immune response against leprosy [14]. Treg and Th17 play roles in the homeostatic of immune system against leprosy and had their own specific roles. Th17 is one of the recently identified effector T cells and acts as proinflammatory cells. Th17 cells have protective properties against leprosy infection using reactive oxygen species by producing inducible Nitric Oxide Synthase (iNOS) to eliminate *M. leprae* [15, 16]. Th17 cells produce IL-17, one of the proinflammatory cytokines, and the expression increased in leprosy cases with better clinical manifestations such as the PB/tuberculoid tuberculoma (TT) type [5, 11]. In the contrary, Treg plays role in regulating the inflammation activity by inhibiting the activation of effector T cells such as Th17 and by activating, proliferating, and recruiting other Treg cells at the injury site through inflammatory and chemokine mediators [16]. The dysregulation of immune system caused accumulation of Tregs found in multibacillary (MB) leprosy [12]. Recent study showed that Treg cells with transcription factor FOXP3+ in the nucleus are involved in downregulation of the immune response [13]. This could be attributed to the increased expression of FOXP3+ Treg cells in the cases with worse clinical manifestations such as borderline (BL)/lepromatous leprosy (LL) type. The close relation between transmission and dysregulation of body immunity prompted the authors to conduct a study on differences in levels of IL-17 and FOXP3+ in leprosy patients in endemic and nonendemic areas, especially in female and pediatric patients.

## 2. Methods

### 2.1. Research Design and Data Collection Techniques

The study performed using cross sectional design in endemic and nonendemic villages of leprosy, Tuban Regency, East Java Province in March 2020. According to the newest East Province Health Profile Data in 2018, Tuban Regency is a district with a high incidence of leprosy, 173 cases or 5.09% of the total cases of leprosy in East Java, with a percentage of pediatric leprosy patients of 5.81% [17].

**Figure 1:**
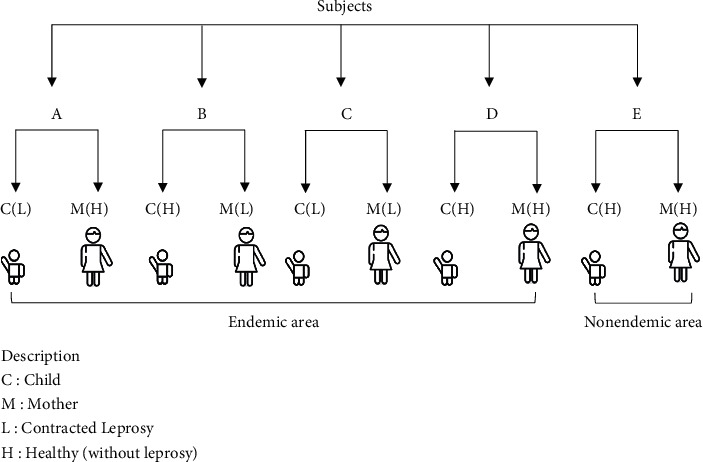
The schematic of subject groupings.

Subjects were divided into 5 groups as follows: group A were children with leprosy (L) and healthy mothers in endemic areas (H); group B are healthy children (H) and mothers with leprosy in endemic areas (L); group C are children with leprosy and mothers with leprosy in endemic areas (L); group D are healthy children and healthy mothers in endemic areas (H); group E are healthy children and healthy mothers in nonendemic areas (H) (Figure 1).

Research data were obtained by taking blood samples and measuring levels of IL-17 and FOXP3+ in the blood circulation using the enzyme-linked immunosorbent assay (ELISA) method. Blood samples were collected from both patients and controls. For ELISA, blood was collected in sterile test tubes and centrifuged for 15 min at 50 g. Serum was separated and kept at −80°C until it's used for estimation of IL-17 and FOXP3+, by the Human IL-17 Quantikinine ELISA Kit and Human FOXP3+ Elisa Kit manufacture guideline. The inclusion and exclusion criteria were used to collect the participants of this study and the number of samples obtained were 82 (41 mothers and 41 children). The inclusion criteria used on this study were (1) productive age/childbearing females aged 20–49 years and children aged 5–18 years, (2) those diagnosed with leprosy, and (3) those who agreed to participate and signed the informed consent in this study. Patients with severe leprosy reactions, pregnant, allergy, autoimmune, or other systemic diseases were excluded from this research. Permission for this research was approved by the Health Research Ethics Committee of Dr. Soetomo, Surabaya (ref. 1664/KEPK/XI/2019.)

### 2.2. Statistical Analysis

Data were processed using Microsoft Excel spread sheets and analyzed using IBM SPSS software (IBM Corp., Armonk, NY, USA). Descriptive statistics and cross tabulation were used to present the data. Comparisons between groups were carried out using the ANOVA test. A *pvalue* <0.05 was considered statistically significant.

## 3. Results

**Figure 2:**
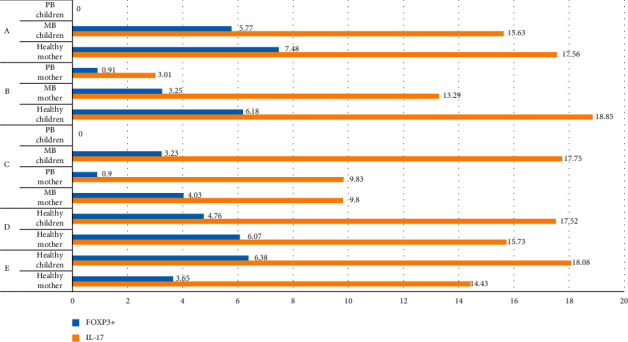
Mean results of IL-17 and FOXP3+ levels.

**Figure 3:**
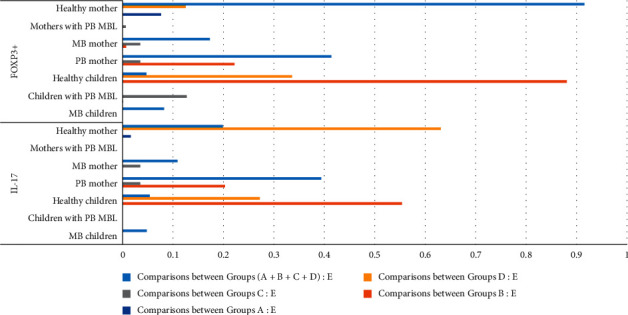
The comparisons of *p* values between groups chart.

This study was conducted in Tuban Regency, East Java Province in March 2020. Total samples obtained were 82 (41 mothers and 41 children). The blood samples obtained to measure the levels of IL-17 and FOXP3+ using ELISA method. The results of this research are explained in Table 1 (summarized in Figure 2) and Table 2 (summarized in Figure 3).

## 4. Discussion

Based on the results of this study, we found a statistically significant difference in IL-17 levels between the group of MB children on endemic areas and healthy children on nonendemic areas (*p*=0.048). Based on the status of leprosy between mother and child patients in endemic areas, significant results were also obtained in MB leprosy children groups A and C with the control group (healthy patients in nonendemic areas), MB leprosy children groups A and C with the control group, PB leprosy mothers in group C with the control group, MB leprosy mothers in groups B and C with the control group, MB leprosy mothers in groups B and C with the control group, and healthy mother group A with control group.

The results of the IL-17 level in this study are similar to a previous study by Sadhu et al. which proved that Th17 was more significantly found in BT/TT type leprosy patients compared to BL/LL (*p* < 0.05), where the levels of IL-17 are higher in patients with PB leprosy than multibacillary (MB) leprosy [12]. This is in line with the findings of our study, where control group consistently showed higher IL-17 levels and significant differences were found in the PB leprosy patient group (mother), MB leprosy patient (child and mother), MB leprosy patient (child and mother), and healthy mother compared to the control group. IL-17 found in leprosy patients can be associated with the inflammatory activities that occur during the leprosy infection process. Proinflammatory cytokines such as TNF-*α*, IL-6, and iNOS which are induced by IL-17 will help the production of reactive oxygen intermediates which contributes to the destruction of *M. leprae* [16]. The significant difference between healthy mother with leprosy children (group A) and control group in this study, where lower IL-17 levels observed in the healthy mother group A, showed that close contact with leprosy patients could increase the risk factors of *M. leprae* infection caused by dysregulation of immune system. As the previous study stated that population with leprosy contacts has 5–8 times higher probability to be infected [5].

In addition, this study also found a significant difference between FOXP3+ levels in healthy children in endemic areas and nonendemic areas (*p*=0.047). Significant results were also found in MB leprosy children in groups A and C with the control group, MB leprosy children in groups A and C with the control group, PB leprosy mothers in group C with the control group, MB leprosy mothers in groups B and C with the control group, and PB MB leprosy mothers in groups B and C with the control group.

Increased expression of FOXP3+ Treg in leprosy cases is related to the suppression of the body's immune system against infection. This is also correlated to the appearance of worse clinical manifestations in people with leprosy. Regulation of the inflammatory reaction is necessary to reduce tissue damage and prevent overactivation of the immune system. In the context of leprosy, Treg is considered to play a role in maintaining the balance of Th1 and Th2 responses. Increased expression of FoxP3+ is often seen in lepromatous leprosy, as well as in erythema nodosum leprosum (ENL) type leprosy reactions^.^ [14, 16]. Treg through the production of cytokines IL-10 and TGF-*α* which is accompanied by an increase in the expression of Programmed Death-1 (PD-1) and its ligands (PD-L1) will shift the Th1 immune response to the tuberculoid form, towards the Th2 immune response in the lepromatous form, and worse clinical manifestation. In the contrary, Th17 through the production of cytokines IL-17A, IL-17F, IL-17A-F, and IL22 will shift the Th2 immune response in the lepromatous form, towards the Th1 immune response in the tuberculoid form [9]. This explains the significant difference in FOXP3+ levels in leprosy patients in endemic areas and healthy patients in nonendemic areas, where lower FOXP3+ is consistently found in the healthy mother populations in nonendemic areas.

The significant difference in FOXP3+ levels shown in this study between healthy children on endemic areas and healthy children in nonendemic areas in this study's findings may indicate the susceptibility of healthy children in endemic areas to suffer from leprosy due to immune dysregulation. In MB/LL leprosy patients, the immune response of leprosy patients dominated by Th2 in response to the damage is caused by the Th1 (CD8+) immune response. This Th2 (CD4+) predominance results in poor outcome and supports the survival of *M. leprae* through inactivation of the macrophage microbicidal response. It was stated that cytokines such as IL-4 supported the survival of *M. leprae* [16]. In MB leprosy patients, Treg (CD4+CD25+) is thought to play an unresponsive role in dealing with *M. leprae* (*M. leprae* specific unresponsiveness) [7, 8].

The unsignificant difference in IL-17 and FOXP3+ was found between healthy populations (mother and children) on leprosy-endemic areas (group D) and healthy populations on nonendemic areas (group E) in this study. These results showed that endemicity of the areas did not contribute to the dysregulation of the immune system directly. The dysregulation of the immune system of the leprosy patients are related to poor nutritional status, health services failure to reduce leprosy stigma, low vaccination number, low nutritional status, not proper breastfeeding, and low environmental hygiene and sanitation including clean water facilities, type of floor, humidity, intensity of sunlight, ventilation, and residential aspects that could be found in several places of endemic areas [6, 7, 18].^.^

## 5. Conclusions and Suggestions

This study found a significant difference in IL-17 levels in children with MB leprosy in endemic areas compared to healthy groups in nonendemic areas. The increased IL-17 in leprosy patients can be associated with the inflammatory activity that occurs during the leprosy infection process. Significant differences were also found in FOXP3+ levels in healthy children in endemic areas compared with healthy groups in nonendemic areas. The findings of this study may indicate the susceptibility of healthy children in endemic areas to suffer leprosy due to immune dysregulation. Endemicity of the areas is not related directly to the dysregulation of the immune system. The factors related to the dysregulation of the immune system such as poor health and environmental factors were found in endemic areas. We suggested more research in the future to investigate the Th1, Th2, and other T cells subset activities, roles, and mechanism in causing the dysregulation of immune system in leprosy disease.

## Figures and Tables

**Table 1:** The mean levels of FOXP3+ and IL-17.

Levels	A			B			C				D		E	
	PB children	MB children	Healthy mother	PB mother	MB mother	Healthy children	PB children	MB children	PB mother	MB mother	Healthy children	Healthy mother	Healthy children	Healthy mother
FoxP3+	0	5.77 ± 2.72	7.48 ± 4.85	0.91 ± 1.52	3.25 ± 3.47	6.18 ± 2.80	0	3.23 ± 1.87	0.90 ± 1.27	4.03 ± 5.69	4.76 ± 2.30	6.07 ± 3.15	6.38 ± 5.28	3.65 ± 1.98
IL-17	0	15.63 ± 2.90	17.56 ± 2.66	3.01 ± 6.37	13.29 ± 7.81	18.85 ± 2.71	0	17.75 ± 2.55	9.83 ± 13.89	9.80 ± 13.86	17.52 ± 2.23	15.73 ± 2.19	18.08 ± 2.73	14.43 ± 2.16

**Table 2:** The comparisons of *p* values between groups.

Variables	Comparisons between groups				
	A: E	B: E	C: E	D: E	(A + B + C + D): E
*IL-17*					
PB children	*p*=.^*a*^		*p*=.^*a*^		*p*=.^*a*^
MB children	*p* < 0.001		*p* < 0.001		*p*=0.048
Children with PB MBL	*p* < 0.001		*p* < 0.001		
Healthy children		*p*=0.554		*p*=0.272	*p*=0.054
PB mother		*p*=0.203	*p*=0.035		*p*=0.394
MB mother		*p* < 0.001	*p*=0.035		*p*=0.109
Mothers with PB MBL		*p* < 0.001	*p* < 0.001		
Healthy mother	*p*=0.016			*p*=0.631	*p*=0.199
*FOXP3+*					
PB children	*p*=.^*a*^		*p*=.^*a*^		*p*=.^*a*^
MB children	*p* < 0.001		*p* < 0.001		*p*=0.082
Children with PB MBL	*p* < 0.001		*p*=0.127		
Healthy children		*p*=0.881		*p*=0.336	*p*=0.047
PB mother		*p*=0.222	*p*=0.035		*p*=0.414
MB mother		*p*=0.007	*p*=0.035		*p*=0.173
Mothers with PB MBL		*p* < 0.001	*p*=0.006		
Healthy mother	*p*=0.076			*p*=0.125	*p*=0.916

## Data Availability

The Microsoft Excel data used for this study are available from the corresponding author upon request.
